# Umbilical cord mesenchymal stem cell transplantation in active and refractory systemic lupus erythematosus: a multicenter clinical study

**DOI:** 10.1186/ar4520

**Published:** 2014-03-25

**Authors:** Dandan Wang, Jing Li, Yu Zhang, Miaojia Zhang, Jinyun Chen, Xia Li, Xiang Hu, Shu Jiang, Songtao Shi, Lingyun Sun

**Affiliations:** 1 Department of Rheumatology and Immunology, the Affiliated Drum Tower Hospital of Nanjing University Medical School, 321 Zhongshan Road, Nanjing, Jiangsu 210008, China; 2 Department of Rheumatology, the Affiliated Hospital of Jiangsu University, 438 Jiefang Road, Zhenjiang 212001, China; 3 Department of Rheumatology, Subei People’s Hospital of Jiangsu Province, 98 Nantong West Road, 225001 Yangzhou, China; 4 Department of Rheumatology, Jiangsu Provincial People’s Hospital, 300 Guangzhou Road, 210029 Nanjing, China; 5 Stem Cell Center of Jiangsu Province, Taizhou, China; 6 Center for Craniofacial Molecular Biology, School of Dentistry, University of Southern California Los Angeles, Los Angeles, CA, USA

## Abstract

**Introduction:**

In our present single-center pilot study, umbilical cord (UC)–derived mesenchymal stem cells (MSCs) had a good safety profile and therapeutic effect in severe and refractory systemic lupus erythematosus (SLE). The present multicenter clinical trial was undertaken to assess the safety and efficacy of allogeneic UC MSC transplantation (MSCT) in patients with active and refractory SLE.

**Methods:**

Forty patients with active SLE were recruited from four clinical centers in China. Allogeneic UC MSCs were infused intravenously on days 0 and 7. The primary endpoints were safety profiles. The secondary endpoints included major clinical response (MCR), partial clinical response (PCR) and relapse. Clinical indices, including Systemic Lupus Erythematosus Disease Activity Index (SLEDAI) score, British Isles Lupus Assessment Group (BILAG) score and renal functional indices, were also taken into account.

**Results:**

The overall survival rate was 92.5% (37 of 40 patients). UC-MSCT was well tolerated, and no transplantation-related adverse events were observed. Thirteen and eleven patients achieved MCR (13 of 40, 32.5%) and PCR (11 of 40, 27.5%), respectively, during 12 months of follow up. Three and four patients experienced disease relapse at 9 months (12.5%) and 12 months (16.7%) of follow-up, respectively, after a prior clinical response. SLEDAI scores significantly decreased at 3, 6, 9 and 12 months follow-up. Total BILAG scores markedly decreased at 3 months and continued to decrease at subsequent follow-up visits. BILAG scores for renal, hematopoietic and cutaneous systems significantly improved. Among those patients with lupus nephritis, 24-hour proteinuria declined after transplantation, with statistically differences at 9 and 12 months. Serum creatinine and urea nitrogen decreased to the lowest level at 6 months, but these values slightly increased at 9 and 12 months in seven relapse cases. In addition, serum levels of albumin and complement 3 increased after MSCT, peaked at 6 months and then slightly declined by the 9- and 12-month follow-up examinations. Serum antinuclear antibody and anti-double-stranded DNA antibody decreased after MSCT, with statistically significant differences at 3-month follow-up examinations.

**Conclusion:**

UC-MSCT results in satisfactory clinical response in SLE patients. However, in our present study, several patients experienced disease relapse after 6 months, indicating the necessity to repeat MSCT after 6 months.

**Trial registry:**

ClinicalTrials.gov identifier: NCT01741857. Registered 26 September 2012.

## Introduction

Systemic lupus erythematosus (SLE) is a common and potentially fatal autoimmune disease characterized by autoantibodies associated with multiorgan injury, including the renal, cardiovascular, neural, musculoskeletal and cutaneous systems [1]. Although disease severity and organ involvement vary significantly among SLE patients, abnormalities of T and B lymphocytes are universal [2-4]. A deeper understanding of the underlying pathology is crucial to the development of optimal therapies for the restoration of immune homeostasis [5].

In addition to conventional immunosuppressive therapies, such as cyclophosphamide (CYC) and mycophenolate mofetil (MMF), several new strategies have been developed to target specific activation pathways relevant to SLE pathogenesis [6]_._ For instance, B-cell-depleting therapies using the monoclonal antibodies rituximab and the B-lymphocyte stimulator (BLyS) inhibitor belimumab have been beneficial in a specific subpopulation of lupus patients [7,8]. Recently, hematopoietic stem cell transplantation (HSCT) has been reported to improve disease activity in treatment-refractory SLE [9] and in reverse organ dysfunction in several animal models [10], but the rates of relapse and treatment-related toxicity are high, as are the rates for the development of a secondary autoimmune disorder [11]_._

Mesenchymal stem cells (MSCs) have been widely studied as an alternative cell source for their ability to differentiate into multiple mesenchymal lineages, including bone, fat and cartilage [12]. Recent studies have indicated that these pluripotent cells can also differentiate into endoderm and neuroectoderm lineages, including neurons, hepatocytes and cardiocytes [13-15]. MSCs have been found to possess immunomodulatory effects on various activated immune cells, such as T cells, B cells, natural killer cells and dendritic cells [16-18]. Additionally, MSCs are able to escape alloantigen recognition because of their low immunogenicity and accompanying lack of expression of costimuatory molecules. These properties make MSCs promising candidate cells for preventing rejection in organ transplantation and treatment of autoimmune disease.

In recent years, we have published pilot single-center clinical studies in which we have reported the safety and efficacy of allogeneic bone marrow– or umbilical cord (UC)–derived MSCs in treating drug-resistant SLE patients, and the clinical results have been encouraging [19,20]. However, we had some relapsed cases during long-term follow-up [21]; thus, we found it is necessary to conduct a multicenter clinical study to further confirm the efficacy of MSC-based treatment and to explore the best effective time to initiate it in lupus patients. In our present multicenter clinical study, we found that intravenous UC MSC transplantation (MSCT) was safe and observed no transplantation-related adverse events. UC MSC treatment resulted in clinical disease remission and systemic amelioration in lupus patients who are refractory to other. However, some patients had disease relapses after 6 months; therefore, we believe that a repeated MSC infusion is feasible and necessary after 6 months to avoid disease relapse.

## Methods

### Patients

From December 2009 to August 2011, 40 SLE patients ranging in age from 17 to 54 years were enrolled into our trial. Informed consent was obtained from each patient and donor. All enrolled patients met at least four of the eleven American College of Rheumatology criteria for SLE. The eligibility criteria included treatment-refractory and active disease, as well as a Systemic Lupus Erythematosus Disease Activity Index (SLEDAI) score of more than 8 or at least one British Isles Lupus Assessment Group (BILAG) grade A or at least two BILAG grade B manifestations. *Refractory to treatment* was defined as lack of response to treatment with monthly intravenous pulse CYC (500 to 750 mg/m^2^) for at least 6 months [22,23], or lack of response to treatment with oral MMF (≥ 1,000 mg/day) [24] or leflunomide (20 mg/day) for at least 3 months, or continued daily doses of at least 20 mg of prednisone (Pred) or its equivalent. Patients were excluded from the study if they had uncontrolled infection, New York Heart Association functional classification III or IV, failure of one of the vital organs or were pregnant or lactating. *Active lupus nephritis* (LN) was defined by meeting at least one of the following criteria: (1) laboratory tests documenting active LN three consecutive times: decrease in renal function (serum creatinine > 1.2 mg/dl), increase in proteinuria (> 1.0 g of protein excretion in a 24-hour urine specimen), deterioration in microscopic hematuria (> 10 red blood cells per high-power field) or the presence of cellular casts; or (2) renal biopsy documenting LN according to the International Society of Nephrology/Renal Pathology Society 2003 classification system criteria for active or active/chronic LN in renal biopsy class III, class IV-S or class IV-G, class V, class III + class V or class IV + class V [25]. The study was conducted in compliance with current good clinical practice (GCP) standards and in accordance with the principles set forth under the 1989 Declaration of Helsinki. The protocol was approved by the Ethics Committee at The Drum Tower Hospital of Nanjing University Medical School, The Affiliated Hospital of Jiangsu University, Jiangsu Provincial People’s Hospital and Subei People’s Hospital of Jiangsu Province.

### Study design

UC MSCs were prepared by the Stem Cell Center of Jiangsu Province, which is the National Stem Cell Institute in China and a member of the International Society for Cellular Therapy. The Stem Cell Center was also certified by the American Association of Blood Banks. Fresh UCs were obtained from informed healthy mothers in a local maternity hospital after normal deliveries. The UCs were rinsed twice in phosphate-buffered saline in penicillin and streptomycin, and the cord blood was removed during this process. The washed UCs were cut into 1-mm^2^ pieces and floated in low-glucose Dulbecco’s modified Eagle’s medium containing 10% fetal bovine serum. The pieces of UC were subsequently incubated at 37°C in a humidified atmosphere consisting of 5% CO_2_. Nonadherent cells were removed by washing. The medium was replaced every 3 days after the initial plating. When well-developed colonies of fibroblast-like cells appeared after about 10 days, the cells were trypsinized and passaged into a new flask for further expansion.

Cell viability was determined by trypan blue testing. The culture supernatant was analyzed for pathogenic microorganisms by direct cultivation analysis. Supernatant levels of alanine aminotransferase and endotoxins for each cell preparation were determined using an automatic biochemistry analyzer and by tachypleus amebocyte lysate analysis, respectively. In addition, supernatant virus indexes were determined by enzyme-linked immunosorbent assay. Cell surface labeling markers, including CD29, CD73, CD90, CD105, CD45, CD34, CD14, CD79 and human leukocyte antigen major histocompatibility complex class II molecule, DR haplotype (HLA-DR), as well as their isotype controls, were all purchased from eBioscience (San Diego, CA, USA), and cell phenotypes were studied by flow cytometric analysis (FCM). We used good manufacturing practice conditions and clinical grade reagents to prepare the cells, and the protocol was conducted in compliance with GCP standards. One million cells per kilogram of body weight were administered by intravenous infusion on days 0 and 7.

### Endpoints

Each patient returned for follow-up at 1, 3, 6, 9 and 12 months after MSCT. Evaluations performed at these follow-up visits included a physical examination, determination of SLEDAI score, BILAG analysis, serologic studies and evaluation of organ function. Adverse events and their severity were assessed and recorded throughout the study. Primary efficacy endpoints were major clinical response (MCR) and partial clinical response (PCR) assessed during the 12-month study period. A MCR was defined as achieving BILAG C scores or better in all organs at 6 months without experiencing a severe flare, which was defined, in turn, as one new domain with a BILAG A score or two new domains with BILAG B scores from MSC infusion and maintenance of this response throughout the 12-month study period. A PCR was defined as (1) BILAG C scores or better and maintenance of this response without a new BILAG A or B score within 3 months; and (2) having no more than one organ with a BILAG B score at 6 months without achieving at least one new BILAG A or B score throughout the 12-month study period [26]. *No clinical response* was defined as failure to meet the definition of a MCR or PCR. Clinical relapse was defined as development of at least one new domain with a BILAG A or B score after a previous MCR or PCR. Secondary efficacy endpoints included SLEDAI score, lupus serologic changes, systemic evaluations such as renal functional indexes, and hematological involvement. Transplantation-related mortality included all deaths associated with UC MSCT, except those related to recurrence of underlying disease. The investigators assessed and recorded adverse events and their severity throughout the study.

After UC MSCT, the doses of steroids as well as immunosuppressive drugs were tapered according to the amelioration of disease conditions. The dose of Pred was tapered by 5 to 10 mg every 2 weeks during the first month following transplantation for responders. If the clinical index was not improved or if disease activity had not declined, which was defined as nonresponse, the drug dose was not tapered or new drugs might be chosen. When relapse occurred, the dose of Pred or immunosuppressive drug would be added or new drugs would be given. This protocol is uniformly adhered to at each center, and the trial was monitored by the third party (The Stem Cell Center of Jiangsu Province).

### Statistical analysis

Data were analyzed as of the last data collection in August 2011. Patients were censored at the time of death or last follow-up. We used Fisher’s exact test to compare the distribution of categorical variables. Pairwise comparisons of pre- and post-MSCT variables were analyzed by paired *t*-test analysis using SPSS version 13.0 statistical software (IBM SPSS, Chicago, IL, USA). The comparisons of clinical responses between patients with or without CYC treatment were analyzed by χ^2^ test. The BILAG index for different organ systems was used to assess response, and scores were converted to numeric values (A = 9, B = 3, C = 1, D = 0 and E = 0) to enable evaluation [27,28]. All *P* values were two-sided, and *P* < 0.05 was considered statistically significant.

## Results

### Participant characteristics

Forty patients, including thirty-eight females and two males, were enrolled in this trial. Twenty-six patients were enrolled from The Department of Rheumatology, the Affiliated Drum Tower Hospital of Nanjing University Medical School, Nanjing, China, 6, 5 and 3 patients were enrolled from The Department of Rheumatology, the Affiliated Hospital of Jiangsu University, Zhenjiang, China, the Department of Rheumatology, Subei People’s Hospital of Jiangsu Province, Yangzhou, China, and the Department of Rheumatology, Jiangsu Provincial People’s Hospital, Nanjing, China, respectively. The mean disease duration was 90.9 months, ranging from 15 to 264 months. Baseline demographics and clinical manifestations for each patient are shown in Table 1. Thirty-nine patients (39/40, 97.5%) underwent two times of UC MSC infusions with an interval of one week, and one patient (1/40, 2.5%) was exempted from the second MSC infusion because of uncontrolled disease progression.

**Table 1:** **Clinical manifestation for each patient at baseline**^**a **^(n = 40)*

**Patient/age (yr)**	**Disease duration (mo)**	**Baseline SLEDAI score**	**Baseline BILAG score**	**Total cumulative dose of IS**	**Clinical outcomes after MSCT**	**Clinical manifestations**
1/46	40	17	12	CYC 0.8 gm/mo × 28 mo	PCR	LN, A, C, V, H, ANA+, anti-dsDNA+
2/37	41	12	12	CYC 0.8 gm/mo × 35 mo	PCR	A, LN, V, ANA+, anti-dsDNA+, H
3/21	50	11	9	MMF 1.5 gm/d × 31 mo	NR	V, LN, C, anti-SM+
4/28	98	9	9	CYC 0.8 gm/mo × 10 mo, CYC 0.8 gm/mo combined with MMF 1.0 gm/d × 28 mo (discontinue), LEF 20 mg/d × 31 mo	MCR	V, A, alopecia, LN, C, ANA+, anti-dsDNA+
5/26	120	12	8	MMF 2.0 gm/d × 50 mo (discontinue), CYC 0.8 gm/mo × 20 mo	NR	V, A, LN, ANA+, anti-dsDNA+
6/23	15	14	19	CYC 0.8 gm/mo × 15 mo, LEF 20 mg/d × 10 mo	NR	V, A, F, LN, P, ANA+, anti-dsDNA+
7/20	62	12	18	MMF 1.5 gm/d × 34 mo (discontinued), CYC 0.8 gm/mo × 24 mo	PCR	A, F, LN, C, P, ANA+
8/43	26	34	20	CYC 0.8 gm/mo × 10 mo (discontinued), LEF 20 mg/d × 10 mo	PCR → R	C, V, LN, A, seizures, ANA+
9/36	97	10	26	CYC 0.8 gm/mo × 29 mo	MCR → R	C, V, A, LN, P, ANA+
10/39	60	10	7	CYC 0.8 gm/mo × 25 mo (discontinued), LEF 20 mg/d × 30 mo	PCR	LN, A, V, ANA+, anti-SM+
11/22	40	8	16	CYC 0.8 gm/mo × 25 mo	NR	LN, C, P, ANA+, anti-dsDNA+
12/20	50	14	13	CYC 0.8 gm/mo × 15 mo (discontinued), VCR 1 mg/week × 4times (discontinued)	NR	A, severe thrombocytopenia, V, F, ANA+, anti-dsDNA+, anti-SM+
13/17	75	7	6	MMF 1.5 gm/d × 13 mo (discontinued), LEF 20 mg/d × 30 mo	NR	Severe thrombocytopenia, LN, A, ANA+, anti-dsDNA+
14/21	39	12	11	CYC 0.8 gm/mo × 17 mo	NR	LN, F, P, A, anti-dsDNA+
15/36	60	10	7	LEF 20 mg/d × 20 mo (discontinued), CYC 0.8 gm/mo × 37 mo	MCR	LN, V, P, A, ANA+, anti-SM+
16/16	49	11	15	CYC 0.8 gm/mo × 17 mo (discontinued), LEF 20 mg/d × 20 mo	NR	LN, A, V, ANA+
17/44	145	4	8	CYC 0.8 gm/mo × 64 mo	NR	LN, A, V, C, ANA+
18/44	85	8	9	CYC 0.8 gm/mo × 40 mo	PCR	A, LN, F, ANA+, anti-dsDNA+
19/29	86	10	5	CYC 0.8 gm/mo × 24 mo	PCR → R	LN, A, P, F, ANA+, anti-dsDNA+
20/54	264	8	4	CYC 0.8 gm/mo × 36 mo (discontinued), MMF 1.5 gm/d × 12 mo, then MMF 1.0 gm/d × 28 mo	MCR	LN, A, V, C, ANA+
21/36	121	13	13	CYC 0.8 gm/mo × 25 mo, LEF 20 mg/d × 40 mo	PCR → R	LN, A, V, C
22/40	24	12	8	CYC 0.8 gm/mo × 18 mo	NR	F, V, LN, C, ANA+
23/35	25	14	24	CYC 0.8 gm/mo × 21 mo	NR	F, A, V, LN, P
24/27	48	12	7	LEF 20 mg/d × 4 mo (discontinued), CYC 0.8 gm/mo × 40 mo	MCR	LN, F, A, P, ANA+, anti-SM+
25/30	102	10	7	MMF 2.0 gm/d × 6 mo, then tapered to 1.5 gm/d × 36 mo, LEF 20 mg/d × 12 mo, then tapered to 10 mg/d × 59 mo	PCR	V, A, LN, ANA+, anti-dsDNA+
26/31	62	8	3	MMF 1.5 gm/d × 8 mo, then tapered to 1.0 gm/d × 50 mo, LEF 20 mg/d × 19 mo	MCR → R	LN, V, P, ANA+, anti-dsDNA+
27/51	108	13	29	CYC 0.8 gm/mo × 41 mo, CsA 150 mg/d × 30 mo	NR	LN, V, A, C, seizures
28/50	110	10	11	LEF 20 mg/d × 39 mo (discontinued), CYC 0.8 gm/mo × 36 mo	MCR	A, V, LN, ANA+
29/45	102	10	9	CYC 1.2 gm/mo × 22 mo	NR	A, V, LN, ANA+, anti-dsDNA+
30/33	62	10	9	CYC 0.8 gm/mo × 21 mo, LEF 20 mg/d × 12 mo	MCR	LN, A, P, C
31/32	156	14	12	CYC 0.8 gm/mo × 36 mo (discontinued), MMF 1.5 gm/d × 6 mo, then tapered to 1.0 gm/d × 56 mo	MCR → R	LN, A, C, P
32/53	146	12	10	CYC 0.8 gm/mo × 24 mo, then tapered to 0.6 gm/mo × 42 mo, LEF 20 mg/d × 12 mo, then tapered to 10 mg/d × 40 mo	NR	LN, A, C, anti-dsDNA+
33/30	157	8	7	CYC 0.8 gm/mo × 16 mo (discontinued)	NR	V, LN, A, C, H
34/35	123	10	9	CYC 0.8 gm/mo × 18 mo, LEF 20 mg/d × 22 mo	NR	LN, A, H, C, ANA+
35/33	216	10	3	CYC 0.8 gm/mo × 26 mo	PCR	F, LN, V, C, A
36/39	99	5	7	CYC 0.8 gm/mo × 14 mo	MCR	LN, C, V, anti-dsDNA+
37/35	109	6	6	LEF 20 mg/d × 34 mo	PCR → R	LN, C, H, ANA+
38/31	160	9	7	LEF 20 mg/d × 13 mo (discontinued), CYC 0.8 gm/mo × 35 mo	MCR	LN, C, V, A
39/50	108	10	12	CYC 0.8 gm/mo × 14 mo	MCR	F, LN, A, V, C
40/35	96	8	8	CYC 0.8 gm/mo × 28 mo (discontinued), LEF 20 mg/d × 7 mo	MCR	LN, V, P, C, ANA+

^a^A, Arthralgia; ANA, Antinuclear antibody; Anti-dsDNA, anti-double-stranded DNA antibody; C, Cytopenia; F, Febrile; H, Hypocomplementemia; IS, Immunosuppressive drug; LN, Lupus nephritis; MCR, Major clinical response; NR, Nonresponse; P, Polyserositis; PCR, Partial clinical response; R, Relapse; V, Vasculitis; VCR, Vincristine. Patients’ clinical manifestations were recorded within 1 week before umbilical cord mesenchymal stem cell transplantation. Total patient population = 40. Patients 1 to 26 were enrolled from the Department of Rheumatology, Affiliated Drum Tower Hospital, Nanjing University Medical School, Nanjing, China. Patients 27 to 32 were enrolled from the Department of Rheumatology, Affiliated Hospital of Jiangsu University, Zhenjiang, China. Patients 33 to 37 were enrolled from the Department of Rheumatology, Subei People’s Hospital of Jiangsu Province, Yangzhou, China. Patients 38 to 40 were enrolled from the Department of Rheumatology, Jiangsu Provincial People’s Hospital, Nanjing, China.

### Umbilical cord mesenchymal stem cell characteristics

All the infused UC MSCs were derived from passages 2 to 4, with rigorous purification and quality control. The cell viability of purified MSCs was greater than 92%. The culture supernatant was negative for pathogenic microorganisms, including aerobic and anaerobic bacteria, as well as for hepatitis B surface antigen, hepatitis B core antibody, hepatitis C virus antibody, HIV antibodies I and II, cytomegalovirus immunoglobulin M and syphilis antibody. FCM analysis showed CD29, CD73, CD90 and CD105 expression greater than 95% in parallel with CD45, CD34, CD14, CD79 and HLA-DR expression less than 2%. In addition, levels of alanine aminotransferase and endotoxins in the supernatants of each cell preparation were strictly controlled within 40 IU/L and 5 endotoxin units, respectively. The capacity of MSCs to differentiate into adipogenic and osteogenic lineages was also assayed.

### Safety

After 12 months, the overall survival rate was 92.5% (37 of 40 patients). Three patients died as a result of uncontrolled disease activity and organ failure. One patient had active lupus with malar rash, arthralgia, uncontrolled hypertension and rapid deterioration of renal function, hypoproteinemia and severe proteinuria. She died 7 days after the first MSC infusion as a result of uncontrolled progressive disease and acute heart failure. Another patient had a lupus relapse 8 months after MSC infusion, with pulmonary hypertension, and died as a result of right-sided heart failure 256 days after MSCT. The third patient also had disease relapse 6 months after MSCT, with steroid-resistant thrombocytopenia and uncontrolled septicemia, and ultimately died due to respiratory failure 192 days after MSC infusion. Two patients had moderate herpesvirus infection 291 and 135 days after MSC treatment, respectively, and one patient had tuberculosis infection at 326 days. All the infection adverse events were treated by conventional therapies. Adverse events were not considered to be possibly related to UC MSCT. All the adverse events are listed in Table 2.

**Table 2:** **Adverse events by umbilical cord mesenchymal stem cell treatment within 12 months**^**a**^

**Patient**	**Adverse events**	**Severity**	**Time (days)**	**Related to MSCT**
2	Herpesvirus infection	AE	291	No relation
3	Herpesvirus infection	AE	135	No relation
3	Herpesvirus infection	AE	187	No relation
9	Death	SAE	7	No relation
12	Tuberculosis infection	AE	326	No relation
14	Death	SAE	256	No relation
27	Death	SAE	192	No relation

^a^AE, Adverse event; MSCT, Mesenchymal stem cell treatment; SAE, Severe adverse event.

### Clinical outcomes

#### *Clinical responses*

Thirteen and eleven patients achieved MCR (13 of 40 patients, 32.5%) and PCR (11 of 40 patients, 27.5%), respectively, during 12 months of follow-up. In total, 16 patients had no clinical response (16 of 40 patients, 40%). Three and four patients, respectively, experienced disease relapse at 9 months (12.5%) and 12 months (16.7%) of follow-up after a prior MCR or PCR. Twenty-six patients received CYC as basal treatment, and the other fourteen patients did not. However, we did not observe any difference in the rate of clinical remission between the two groups (*P* > 0.05 by χ^2^ test).

### Disease activity assessment

**Figure 1:**
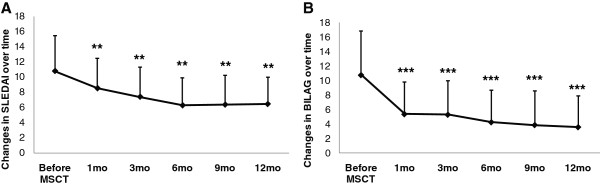
**Graphs illustrate changes in clinical status before and after umbilical cord mesenchymal stem cell transplantation.** Changes in clinical status from before umbilical cord mesenchymal stem cell transplantation (MSCT) and afterward were assessed based on Systemic Lupus Erythematosus Disease Activity Index (SLEDAI) score **(A)** and total British Isles Lupus Assessment Group (BILAG) score **(B)**. **P < 0.01 versus before MSCT. ***P < 0.001 versus before MSCT. Error bars mean SD values.

Lupus disease activity, as defined by SLEDAI score, significantly decreased after MSCT (mean ± SD values = 10.83 ± 4.63 at baseline, 8.55 ± 3.99 at 1 month, 7.43 ± 3.93 at 3 months, 6.30 ± 3.63 at 6 months, 6.40 ± 3.84 at 9 months and 6.48 ± 3.52 at 12 months; all *P* < 0.01 versus baseline levels) (Figure 1A). Total BILAG score was markedly ameliorated after UC MSC infusion (mean ± SD values = 10.78 ± 6.09 at baseline, 5.35 ± 4.48 at 1 month, 5.28 ± 4.71 at 3 months, 4.23 ± 4.43 at 6 months, 3.85 ± 4.73 at 9 months and 3.55 ± 4.33 at 12 months; all *P* < 0.001 versus baseline levels) (Figure 1B).

### Serology changes

**Figure 2:**
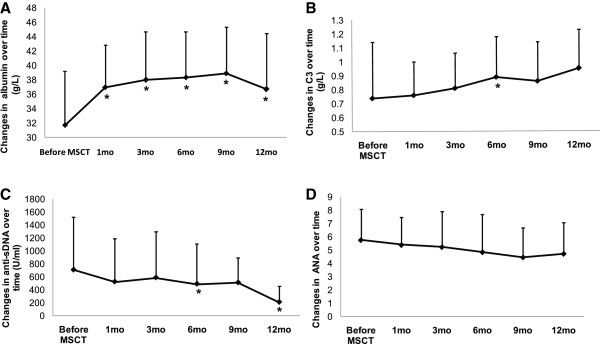
**Graphs illustrate improvement after allogeneic umbilical cord mesenchymal stem cell transplantation.** Improved levels serum albumin **(A)** and complement 3 (C3) **(B)** in lupus patients refractory to other treatments. Serum levels of anti-double-stranded DNA (anti-sDNA) antibody **(C)** and antinuclear antibody (ANA) **(D)** decreased after MSC infusions. *P < 0.05 versus before mesenchymal stem cell transplantation (MSCT). Error bars mean SD values.

Serum albumin levels improved shortly after UC MSC infusions, were normal at the 1-month follow-up visit and remained normal during the succeeding 9 months until the 12-month follow-up visit, when they declined (mean ± SD values = 3.17 ± 0.75 g/dl at baseline, 3.70 ± 0.58 g/dl at 1 month, 3.80 ± 0.67 g/dl at 3 months, 3.84 ± 0.63 g/dl at 6 months, 3.89 ± 0.64 g/dl at 9 months and 3.67 ± 0.78 g/dl at 12 months; all *P* < 0.05 versus baseline levels) (Figure 2A). Serum complement 3 improved with statistical significance found at 6 months (Figure 2B). Serum complement 4 levels showed no obvious changes after MSC treatment in those patients. We observed that serum anti-double-stranded DNA antibody levels decreased after MSCT with statistically significant differences found at the 6- and 12-month follow-up visits (mean ± SD values = 710.83 ± 814.05 U/ml at baseline, 526.78 ± 666.7 U/ml at 1 month, 590.41 ± 702.99 U/ml at 3 months, 492.67 ± 615.15 U/ml at 6 months, 513.58 ± 378.6 U/ml at 9 months and 212.62 ± 244.77 U/ml at 12 months; *n* = 16), along with decreased serum antinuclear antibody (mean ± SD values = 5.77 ± 2.32 at baseline, 5.40 ± 2.08 at 1 month, 5.24 ± 2.66 at 3 months, 4.85 ± 2.83 at 6 months, 4.46 ± 2.21 at 9 months and 4.73 ± 2.36 at 12 months) (Figures 2C and D).

### Organ function improvement

**Figure 3:**
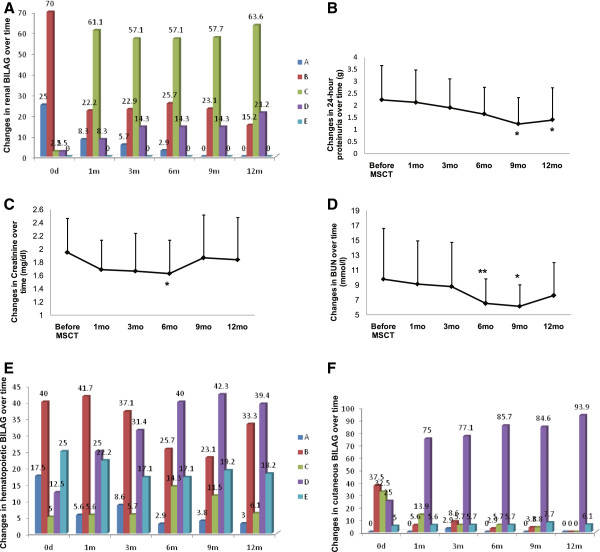
**Graphs illustrate marked improvement in renal system after umbilical cord mesenchymal stem cell transplantation. (A)** British Isles Lupus Assessment Group (BILAG) score improved over time. Twenty-four hours after umbilical cord mesenchymal stem cell transplantation (MSCT), declines were observed in proteinuria **(B)**, serum creatinine **(C)** and blood urea nitrogen (BUN) **(D)**. BILAG scores for the hematopoietic **(E)** and cutaneous **(F)** systems were ameliorated after MSCT. *P < 0.05 versus before MSCT. **P < 0.01 versus before MSCT. Error bars mean SD values.

Thirty-eight (95%) of forty patients had active LN (renal BILAG A or B score) at baseline, but their renal BILAG scores decreased significantly after two UC MSC infusions (Figure 3A). Twenty-four-hour proteinuria levels decreased significantly after UC MSC treatment (mean ± SD values = 2.24 ± 1.43 g at baseline, 2.13 ± 1.35 g at 1 month, 1.91 ± 1.20 g at 3 months, 1.65 ± 1.11 g at 6 months, 1.24 ± 1.09 g at 9 months and 1.41 ± 1.33 g at 12 months; *P* < 0.05 at 9- and 12-month follow-up visits) (Figure 3B). Renal function index, as assessed by serum creatinine and blood urea nitrogen levels, also decreased, and both showed statistically significant differences at the 6-month follow-up visit (Figures 3C and D), but they increased at the 12-month follow-up visit. Twenty-five (62.5%) and twenty-eight (70%) of the forty patients had hematopoietic and cutaneous system involvement, respectively, at baseline. The BILAG score for the two systems also ameliorated after MSC treatment (Figures 3E and F).

### Therapy schedule after umbilical cord mesenchymal stem cell infusion

The dose of Pred was tapered from 5 to 10 mg every 2 weeks during the first month following transplantation, according to clinical status and laboratory indicators of disease amelioration. During the 12-month follow-up visits, 30 (81.08%) of 37 patients underwent steroid tapering, and, though 19 (54.29%) of 35 patients underwent immunosuppressant tapering after MSCT, two patients were excluded because they had not been taking immunosuppressive drugs at baseline (Table 3).

**Table 3:** **Treatments used before and after umbilical cord mesenchymal stem cell transplantation in each patient**^**a**^

**Patient**	**Baseline**	**1 month**	**3 months**	**6 months**	**12 months**
1	Pred 5 mg/d	Pred 5 mg/d,	Pred 5 mg/d	Pred 5 mg/d	Pred 5 mg/d
	CYC 0.8 g/mo	CYC 0.8 g/mo,	CYC 0.8 g/mo	CYC 0.8 g/mo	CYC 0.8 g/mo
	LEF 20 mg/d	LEF 20 mg/d,	LEF 20 mg/d	LEF 20 mg/d	LEF 20 mg/d
	HCQ 200 mg/d	HCQ 200 mg/d	HCQ 200 mg/d	HCQ 200 mg/d	HCQ 200 mg/d
2	Pred 30 mg/d	Pred 15 mg/d	Pred 10 mg/d	Pred 10 mg/d	Pred 10 mg/d
	CYC 0.8 g/mo	CYC 0.8 g/mo	CYC 0.8 g/mo	CYC 0.8 g/mo	CYC 0.8 g/mo
3	Pred 15 mg/d	Pred 15 mg/d	Pred 10 mg/d	Pred 15 mg/d	Pred 15 mg/d
	MMF 1.5 g/d	MMF 1.5 g/d	MMF 1.5 g/d	MMF 1.5 g/d	MMF 1.0 g/d
	HCQ 200 mg/d	HCQ 200 mg/d	HCQ 200 mg/d	HCQ 200 mg/d	HCQ 200 mg/d
4	Pred 10 mg/d	Pred 5 mg/d	Pred 5 mg/d	Pred 5 mg/d	Pred 5 mg/d
	LEF 20 mg/d	LEF 20 mg/d	LEF 20 mg/d	LEF 10 mg/d	LEF 10 mg/d
	HCQ 400 mg/d	HCQ 400 mg/d	HCQ 400 mg/d	HCQ 400 mg/d	HCQ 200 mg/d
5	Pred 10 mg/d	Pred 10 mg/d	Pred 5 mg/d	Pred 5 mg/d	Pred 5 mg/d
	CYC 0.8 g/mo	CYC 0.8 g/mo	CYC 0.8 g/mo	CYC 0.8 g/mo	CYC 0.6 g/mo
	HCQ 400 mg/d	HCQ 400 mg/d	HCQ 400 mg/d	HCQ 400 mg/d	HCQ 400 mg/d
6	Pred 20 mg/d	Pred 20 mg/d	Pred 15 mg/d	Pred 15 mg/d	Pred 15 mg/d
	CYC 0.8 g/mo	CYC 0.8 g/mo	CYC 0.8 g/mo	CYC 0.8 g/mo	CYC 0.8 g/mo
	LEF 20 mg/d	LEF 20 mg/d	LEF 20 mg/d	LEF 20 mg/d	LEF 20 mg/d
	HCQ 400 mg/d	HCQ 400 mg/d	HCQ 400 mg/d	HCQ 400 mg/d	HCQ 400 mg/d
				Triptolide 60 mg/d	Triptolide 60 mg/d
7	Pred 15 mg/d	Pred 10 mg/d	Pred 10 mg/d	Pred 10 mg/d	Pred 10 mg/d
	CYC 0.8 g/mo	CYC 0.8 g/mo	CYC 0.8 g/mo	CYC 0.8 g/mo	CYC 0.6 g/mo
	HCQ 300 mg/d	HCQ 300 mg/d	HCQ 300 mg/d	HCQ 300 mg/d	HCQ 300 mg/d
8	Pred 20 mg/d	Pred 15 mg/d	Pred 15 mg/d	Pred 30 mg/d	Pred 10 mg/d
	LEF 20 mg/d	LEF 20 mg/d	LEF 20 mg/d	LEF 20 mg/d	LEF 20 mg/d
	HCQ 400 mg/d	HCQ 400 mg/d	HCQ 400 mg/d	HCQ 400 mg/d	HCQ 300 mg/d
9	Pred 40 mg/d	Pred 25 mg/d	Pred 15 mg/d	Pred 10 mg/d	/
	CYC 0.8 g/mo	CYC 0.8 g/mo	CYC 0.8 g/mo	CYC 0.8 g/mo	
	HCQ 400 mg/d	HCQ 400 mg/d	HCQ 400 mg/d	HCQ 400 mg/d	
10	Pred 10 mg/d	Pred 5 mg/d	Pred 5 mg/d	Pred 5 mg/d	Pred 5 mg/d
	LEF 20 mg/d	LEF 20 mg/d	LEF 10 mg/d	LEF 10 mg/d	LEF 10 mg/d
	HCQ 400 mg/d	HCQ 400 mg/d	HCQ 400 mg/d	HCQ 400 mg/d	HCQ 400 mg/d
11	Pred 20 mg/d	Pred 20 mg/d	Pred 15 mg/d	Pred 10 mg/d	Pred 10 mg/d
	CYC 0.8 g/mo	CYC 0.8 g/mo	CYC 0.8 g/mo	CYC 0.8 g/mo	CYC 0.4 g/mo
		HCQ 400 mg/d	HCQ 400 mg/d	HCQ 400 mg/d	HCQ 400 mg/d
12	Pred 20 mg/d	Pred 15 mg/d	Pred 15 mg/d	Pred 10 mg/d	Pred 10 mg/d
	HCQ 400 mg/d	HCQ 400 mg/d	HCQ 400 mg/d	HCQ 400 mg/d	HCQ 400 mg/d
13	Pred 20 mg/d	Pred 15 mg/d	Pred 15 mg/d	Pred 10 mg/d	Pred 10 mg/d
	LEF 20 mg/d	LEF 20 mg/d	LEF 20 mg/d	LEF 20 mg/d	LEF 20 mg/d
	HCQ 400 mg/d	HCQ 400 mg/d	HCQ 400 mg/d	HCQ 400 mg/d	HCQ 400 mg/d
14	Pred 15 mg/d	/	/	/	/
	CYC 0.8 g/mo				
15	Pred 15 mg/d	Pred 15 mg/d	Pred 15 mg/d	Pred 10 mg/d	Pred 10 mg/d
	CYC 0.8 g/mo	CYC 0.8 g/mo	CYC 0.8 g/mo,	CYC0.8 g/mo,	CYC 0.4 g/mo
	HCQ 400 mg/d	HCQ 400 mg/d	HCQ 300 mg/d	HCQ 300 mg/d	HCQ 300 mg/d
16	Pred 15 mg/d	Pred 15 mg/d	Pred 10 mg/d	Pred 10 mg/d	Pred 20 mg/d
	LEF 20 mg/d	LEF 20 mg/d	LEF 20 mg/d	LEF 20 mg/d	LEF 20 mg/d
	HCQ 200 mg/d	HCQ 200 mg/d	HCQ 200 mg/d	HCQ 200 mg/d	HCQ 200 mg/d
17	Pred 15 mg/d	Pred 15 mg/d	Pred 15 mg/d	Pred 10 mg/d	Pred 10 mg/d
	CYC 0.8 g/mo	CYC 0.8 g/mo	CYC 0.8 g/45 d	CYC 0.8 g/45d	CYC 0.4 g/mo
	HCQ 200 mg/d	HCQ 200 mg/d	HCQ 200 mg/d	HCQ 200 mg/d	HCQ 200 mg/d
18	Pred 20 mg/d	Pred 15 mg/d	Pred 15 mg/d	Pred 15 mg/d	Pred 10 mg/d
	CYC 0.8 g/mo	CYC 0.8 g/mo	CYC 0.8 g/mo	CYC 0.8 g/mo	CYC 0.8 g/mo
	HCQ 400 mg/d	HCQ 400 mg/d	HCQ 400 mg/d	HCQ 400 mg/d	HCQ 400 mg/d
19	Pred 15 mg/d	Pred 15 mg/d	Pred 10 mg/d	Pred 10 mg/d	Pred 15 mg/d
	CYC 0.8 g/mo	CYC 0.8 g/mo	CYC 0.8 g/mo	CYC 0.6 g/mo	CYC 0.8 g/mo
	HCQ 400 mg/d	HCQ 400 mg/d	HCQ 400 mg/d	HCQ 400 mg/d	HCQ 400 mg/d
20	Pred 5 mg/d	Pred 5 mg/d	Pred 5 mg/d	Pred 5 mg/d	Pred 5 mg/d
	MMF 1.0 g/d	MMF 1.0 g/d	MMF 1.0 g/d	MMF 1.0 g/d	MMF 0.5 g/d
	HCQ 200 mg/d	HCQ 200 mg/d	HCQ 200 mg/d	HCQ 200 mg/d	HCQ 200 mg/d
21	Pred 7.5 mg/d	Pred 7.5 mg/d	Pred 5 mg/d	Pred 15 mg/d	Pred 15 mg/d
	CYC 0.8 g/mo	CYC 0.8 g/mo	CYC 0.8 g/mo	CYC 0.8 g/mo	CYC 0.8 g/mo
	LEF 10 mg/d	LEF 10 mg/d	LEF 10 mg/d	LEF 10 mg/d	LEF 10 mg/d
	HCQ 400 mg/d	HCQ 400 mg/d	HCQ 400 mg/d	HCQ 400 mg/d	HCQ 200 mg/d
22	Pred 20 mg/d CYC 0.8 g/mo HCQ 400 mg/d	Pred 15 mg/d CYC 0.8 g/mo HCQ 400 mg/d	Pred 15 mg/d CYC 0.8 g/mo HCQ 400 mg/d	Pred 10 mg/d CYC 0.8 g/mo HCQ 400 mg/d	Pred 10 mg/d CYC 0.6 g/mo HCQ 400 mg/d
23	Pred 45 mg/d	Pred 15 mg/d	Pred 10 mg/d	Pred 10 mg/d	Pred 10 mg/d
	CYC 0.8 g/mo	CYC 0.8 g/mo	CYC 0.8 g/mo	CYC 0.8 g/mo	CYC 0.6 g/mo
	HCQ 400 mg/d	HCQ 400 mg/d	HCQ 400 mg/d	HCQ 400 mg/d	HCQ 200 mg/d
24	Pred 15 mg/d	Pred 15 mg/d	Pred 10 mg/d	Pred 5 mg/d	Pred 5 mg/d
	CYC 0.8 g/mo	CYC 0.8 g/mo	CYC 0.8 g/mo	CYC 0.8 g/mo	CYC 0.6 g/mo
25	Pred 15 mg/d	Pred 10 mg/d	Pred 10 mg/d	Pred 10 mg/d	Pred 10 mg/d
	MMF 1.5 g/d	MMF 1.5 g/d	MMF 1.5 g/d	MMF 1.0 g/d	MMF 1.0 g/d
	LEF 10 mg/d	LEF 10 mg/d	HCQ 400 mg/d	HCQ 400 mg/d	HCQ 400 mg/d
	HCQ 400 mg/d	HCQ 400 mg/d			
26	Pred 20 mg/d	Pred 15 mg/d	Pred 10 mg/d	Pred 10 mg/d	Pred 20 mg/d
	MMF 1.0 g/d	MMF 1.0 g/d	MMF 1.0 g/d	MMF 1.0 g/d	MMF 1.0 g/d
	LEF 20 mg/d	LEF 20 mg/d	LEF 20 mg/d	LEF 20 mg/d	LEF 20 mg/d
	HCQ 200 mg/d	HCQ 200 mg/d	HCQ 200 mg/d	HCQ 200 mg/d	HCQ 200 mg/d
27	Pred 50 mg/d	Pred 30 mg/d	Pred 15 mg/d	MP 80 mg/d	/
	CYC 0.8 g/mo	CYC 0.8 g/mo	CYC 0.8 g/mo	CYC 0.6 g/d × 3 d	
	CsA 150 mg/d	CsA 150 mg/d	CsA 150 mg/d	γ-globulin 20 g × 3 d	
28	Pred 20 mg/d	Pred 15 mg/d	Pred 15 mg/d	Pred 5 mg/d	Pred 5 mg/d
	CYC 0.8 g/mo	CYC 0.8 g/mo	CYC 0.8 g/mo	CYC 0.8 g/mo	CYC 0.8 g/mo
	HCQ 200 mg/d	HCQ 200 mg/d	HCQ 200 mg/d	HCQ 200 mg/d	HCQ 200 mg/d
29	Pred 30 mg/d	Pred 25 mg/d	Pred 20 mg/d	Pred 10 mg/d	Pred 5 mg/d
	CYC 1.2 g/mo	CYC 1.2 g/mo	CYC 1.2 g/mo	CYC 1.2 g/mo	CYC 0.8 g/mo
				Triptolide 60 mg/d	Triptolide 60 mg/d
30	Pred 25 mg/d	Pred 20 mg/d	Pred 15 mg/d	Pred 10 mg/d	Pred 10 mg/d
	CYC 0.8 g/mo	CYC 0.8 g/mo	CYC0.8 g/mo	CYC0.8 g/mo	CYC0.6 g/mo
	LEF 20 mg/d	LEF 20 mg/d	LEF 20 mg/d	LEF 20 mg/d	LEF 10 mg/d
31	Pred 30 mg/d	Pred 25 mg/d	Pred 25 mg/d	Pred 30 mg/d	Pred 15 mg/d
	MMF 1.0 g/d	MMF 1.0 g/d	MMF 1.0 g/d	MMF 1.0 g/d	MMF 1.0 g/d
32	Pred 25 mg/d	Pred 20 mg/d	Pred 15 mg/d	Pred 15 mg/d	Pred 15 mg/d
	CYC 0.6 g/mo	CYC 0.6 g/mo	CYC 0.6 g/mo	CYC 0.6 g/mo	CYC 0.6 g/mo
	LEF 10 mg/d	LEF 10 mg/d	LEF 10 mg/d	LEF 10 mg/d	LEF 10 mg/d
	HCQ 400 mg/d	HCQ 400 mg/d	HCQ 400 mg/d	HCQ 400 mg/d	HCQ 200 mg/d
33	Pred 25 mg/d	Pred 15 mg/d	Pred 15 mg/d	Pred 35 mg/d	Pred 35 mg/d
34	Pred 15 mg/d	Pred 15 mg/d	Pred 15 mg/d	Pred 15 mg/d	Pred 10 mg/d
	CYC 0.8 g/mo	CYC 0.8 g/mo	CYC 0.8 g/mo	CYC 0.8 g/mo	CYC 0.8 g/mo
	LEF 20 mg/d	LEF 20 mg/d	LEF 10 mg/d	LEF 10 mg/d	LEF 10 mg/d
35	Pred 20 mg/d,	Pred 15 mg/d	Pred 15 mg/d	Pred 10 mg/d	Pred 10 mg/d
	CYC 0.8 g/mo	CYC 0.8 g/mo	CYC 0.8 g/mo	CYC 0.8 g/mo	CYC 0.6 g/mo
36	Pred 25 mg/d	Pred 15 mg/d	Pred 15 mg/d	Pred 10 mg/d	Pred 10 mg/d
	CYC 0.8 g/mo	CYC 0.8 g/mo	CYC 0.8 g/mo	CYC 0.6 g/mo	CYC 0.6 g/mo
	HCQ 400 mg/d	HCQ 400 mg/d	HCQ 400 mg/d	HCQ 300 mg/d	HCQ 300 mg/d
37	Pred 20 mg/d	Pred 15 mg/d	Pred 15 mg/d	Pred 15 mg/d	Pred 20 mg/d,
	LEF 20 mg/d	LEF 20 mg/d	LEF 20 mg/d	LEF 20 mg/d	LEF 20 mg/d
38	Pred 10 mg/d	Pred 10 mg/d	Pred 10 mg/d	Pred 7.5 mg/d	Pred 7.5 mg/d
	CYC 0.8 g/mo	CYC 0.8 g/mo	CYC 0.8 g/mo	CYC 0.8 g/mo	CYC 0.6 g/mo
	HCQ 200 mg/d	HCQ 200 mg/d	HCQ 200 mg/d	HCQ 200 mg/d	HCQ 200 mg/d
39	Pred 20 mg/d	Pred 15 mg/d	Pred 10 mg/d	Pred 10 mg/d	Pred 10 mg/d
	CYC 0.8 g/mo	CYC 0.8 g/mo	CYC 0.8 g/mo	CYC 0.8 g/mo	CYC 0.8 g/mo
	HCQ 200 mg/d	HCQ 200 mg/d	HCQ 200 mg/d	HCQ 200 mg/d	HCQ 200 mg/d
40	Pred 10 mg/d	Pred 10 mg/d	Pred 10 mg/d	Pred 10 mg/d	Pred 5 mg/d
	LEF 20 mg/d	LEF 20 mg/d	LEF 20 mg/d	LEF 20 mg/d	LEF 20 mg/d

^a^CsA Cyclosporine A; CYC, Cyclophosphamide; HCQ, Hydroxychloroquine; LEF, Leflunomide; MMF, Mycophenolate mofetil; MP, methylprednisone; MSCT, Mesenchymal stem cell transplantation; Pred, Prednisone; UC MSC, Umbilical cord–derived mesenchymal stem cells. Patients 1 to 26 were enrolled from the Department of Rheumatology, Affiliated Drum Tower Hospital, Nanjing University Medical School, Nanjing, China. Patients 27 to 32 were enrolled from the Department of Rheumatology, Affiliated Hospital of Jiangsu University, Zhenjiang, China. Patients 33 to 37 were enrolled from the Department of Rheumatology, Subei People’s Hospital of Jiangsu Province, Yangzhou, China. Patients 38 to 40 were enrolled from the Department of Rheumatology, Jiangsu Provincial People’s Hospital, Nanjing, China.

## Discussion

MSCs are multipotent, nonhematopoietic progenitor cells that are currently being explored as a promising new treatment for tissue regeneration. Although their immunomodulatory properties are not yet completely understood, their low immunogenic potential, together with their effects on immune responses, make them a promising therapeutic tool for the treatment of patients with severe and refractory autoimmune diseases. MSCs have already been applied in clinical treatment for acute graft-versus-host disease following allogeneic HSCT [29,30], ischemic cardiomyopathy [31,32] and autoimmune diseases such as systemic sclerosis [33], inflammatory bowel disease [34,35], dermatomyositis/polymyositis [36], rheumatoid arthritis [37], Sjögren’s syndrome [38] and type 1 or type 2 diabetes mellitus [39,40].

To date, to the best of our knowledge, only limited clinical investigations of MSC treatment in lupus patients have been conducted. We previously conducted a small-scale, short-term study of intravenous delivery of UC MSCs [19]. Recently, a larger-scale study of 87 lupus cases and long-term follow-up of 4 years explored the clinical responses to allogeneic MSCT [21]. However, we did not have the evidence of a multicenter study to further confirm the results. Our present multicenter study has substantiated the clinical safety and efficacy of UC MSCT for the treatment of lupus patients, as determined previously in single-center studies. Sixty percent of patients achieved MCR or PCR after 12 months of follow-up, and another 40% had no clinical response. Intravenous infusion of UC MSCs is a safe practice with treatment efficacy in improving renal function and serologic indices. In addition to a significant decline of disease activity as assessed by SLEDAI and BILAG scores, UC MSC infusion ameliorated systemic manifestations in hematopoietic and cutaneous systems.

We previously compared the clinical efficacy of single and double MSC infusions in lupus patients, and the results showed that the treatment efficacy was comparable between the two groups [41]. In our present multicenter study, 39 of the 40 enrolled patients received double UC MSC infusions with a 1-week interval. At 12 months of follow-up, we found that the clinical response rate and safety profile were comparable. The results further indicate that a single infusion is enough in clinical treatment to be effective.

However, the role of MSCs *in vivo* is not permanent. In the present study, 12.5% and 16.7% of patients had disease relapses at 9 and 12 months of follow-up, respectively, after a prior MCR or PCR. Serologic indices, such as serum albumin and complement 3 levels, reverted slightly toward baseline, concomitantly with relapsed renal function indices, on the basis of serum creatinine and blood urea nitrogen levels. On the basis of the safety profile of MSC infusion in clinical applications, our data suggest the necessity of repeating MSC infusions after 6 months in refractory lupus patients.

MSCs can be isolated from many tissues, including bone marrow, UC, UC blood, placenta or adipose tissue. Bone marrow–derived MSCs, both autologous and allogeneic, are widely used in clinical applications. However, an increasing number of recent studies have shown that MSCs from bone marrow are difficult to obtain, have ethical issues and are easily contaminated. Moreover, autologous bone marrow–derived MSCs are functionally abnormal in some disorders such as lupus [42,43], rheumatoid arthritis [44] and systemic sclerosis [45], which may limit their clinical application. UCs fall off after delivery, but they are rich in MSCs. UC MSCs have many advantages over bone marrow MSCs, including easy access, less possibility of contamination and no ethical problems. Furthermore, UC MSCs, in contrast to bone marrow MSCs, do not express tumor-associated fibroblast phenotypes and therefore have no opportunity to grow solid tumors [46]. Moreover, UC MSCs have a higher rate of gene expression related to cell adhesion, morphogenesis, angiogenesis and neurogenesis than UC blood–derived MSCs do [47], and they can accumulate more mineralized matrix than placenta-derived MSCs [48], indicating that UC MSCs may be used as an optimal cell therapy option.

The present study has some limitations. First, 95% of the patients had active LN at the time of study entry, but we cannot provide the pathologic data of the present enrolled patients. Therefore, we do not know whether MSCs can indeed ameliorate renal pathology, aside from the improvements in renal function. Second, this study is not a randomized controlled trial. It lacks a group of patients who received conventional therapies, but not combined with allogeneic MSC infusion. Therefore, the current data provide evidence only that allogeneic MSCT could induce renal remission on the basis of other drugs taken by patients enrolled in this study. Third, because of the differences in patients’ conditions at the time of enrollment, we cannot be sure of the uniformity and standards for quality control between the different centers or different patients. We will consider performing a multicenter randomized controlled study in China to assess the safety and efficacy of MSCT in LN patients to compare the clinical safety and efficacy of combined steroid/MSC treatment and combined steroid/traditional immunosuppressive drug therapy such as CYC. In the forthcoming trial, repeated renal biopsy will be designed to further determine whether MSCT can alleviate renal pathology in LN patients. In addition, we will try to ensure uniformity among the enrolled patients for quality control.

## Conclusions

Our multicenter clinical study illustrates the safety and efficacy of systemic administration of UC MSCs in SLE patients. Moreover, a repeated MSC infusion is feasible and necessary after 6 months to avoid disease relapse.

## Abbreviations

ANA: Antinuclear antibody; anti-dsDNA: Anti-double-stranded DNA antibody; BILAG: British Isles Lupus Assessment Group; BlyS: B-lymphocyte stimulator; CsA: Cyclosporine A; CYC: Cyclophosphamide; HCQ: Hydroxychloroquine; HSCT: hematopoietic stem cell transplantation; LEF: Leflunomide; LN: Lupus nephritis; MCR: Major clinical response; MMF: Mycophenolate mofetil; MSC: Mesenchymal stem cell; MSCT: Mesenchymal stem cell transplantation; PCR: Partial clinical response; Pred: Prednisone; SLE: Systemic lupus erythematosus; SLEDAI: Systemic Lupus Erythematosus Disease Activity Index.

## Competing interests

The authors declare that they have no competing interests.

## Authors’ contributions

DW, was responsible for the study conception and design, data collection and analysis and manuscript writing. JL, YZ, MZ were responsible for the study design, data collection and analysis and manuscript revision. JC was responsible for data analysis and manuscript writing. XL was responsible for the study conception and design, data collection and manuscript drafting. XH was responsible for the study conception and design and critical revision of the manuscript. SJ was responsible for the study conception and design, data collection and manuscript drafting. SS was responsible for the study conception and design and critical revision of the manuscript. LS was responsible for the study conception and design, data collection and analysis, manuscript writing and final approval of the manuscript. All authors read and approved the final manuscript.
